# Supplementary material for: Post-COVID-19 Cognitive Decline and Apoe Polymorphism: Towards a Possible Link?
Source: Brain Sci. 2023 Nov 21;13(12):1611. doi: 10.3390/brainsci13121611 (PMC10742128; doi:10.3390/brainsci13121611)
Supplement: Supplementary file 1 [file brainsci-13-01611-s001.zip › brainsci-2702571-supplementary.pdf]

SUPPLEMENTAL MATERIAL

Cognitive assessment

Participants underwent application of Addenbrooke's Cognitive Examination-Revised (ACE-R), Mini-Mental State Examination (MMSE), and Clinical Dementia Rating (CDR). Pfeffer instrumental activities of daily living scale was applied to assess functionality and the Geriatric Depression Scale (GDS) to assess mood, or the Beck Inventory, depending on patient age.[49-54] Furthermore, the PRMQ scale was applied for retrospective memory assessment.[55] The values of 58, 76, and 83 were used as the cutoff point for the ACE-R respectively for <4, 4-8, and > 8 years of schooling.[56,57] For the MMSE, the cutoff points of 19 and 24, respectively for 0 and up to 4 years of schooling, were used.[58,59] Functional impairment was defined from a score of 3 on the Pfeffer scale.[60] Regarding psychiatric evaluation, we used a cutoff point of 3 on the GDS and ten on the Beck inventory for a depression diagnosis.[54,61] Cognitive impairment was defined, in this study, when there was a cognitive complaint confirmed proven by screening tests, regardless of functional impairment. Patients with cognitive complaints without objective impairment in the tests performed were characterized as subjective cognitive decline.[13]

2.3 APOE genotyping analysis

According to the manufacturer's instructions, the patient's blood samples were collected in EDTA tubes, and subsequently, genomic DNA was extracted from peripheral blood leukocytes with the commercial PureLink™ Genomic DNA Mini Kit® (Invitrogen).[62] ApoE genotypes were determined by real-time Polymerase Chain Reaction (qPCR) using the TaqMan® allelic discrimination system (TaqMan® SNP Genotyping Assay, ThermoFisher®).[14] For this, we used probes according to the sequences provided by the manufacturer: C___3084793_20 (rs429358) and C____904973_10 (rs7412), observing the information contained in the catalog number:

4351379 and similar protocols described in the literature for performing the technique. All analysis was performed in the QuantStudio® 5 real-time PCR platform (Applied Biosystems®, Foster City, CA, USA).[62]

Ethical Aspects

The Research Ethics Committee of the Walter Cantídio University Hospital approved the study project under the number 4.092.933. All patients signed an Informed Consent Form, with the right to privacy and confidentiality of the information obtained, and could refuse to participate in the proposed activities and questions.

Table S1. Sociodemographic, clinical characteristics and post-Covid symptoms in groups without and with cognitive impairment (CI).

	No Cognitive impairment (n=183)	CI (n=36)	p*
**Gender**			0.371
Female	121 (66.1)	21 (58.3)	
Male	62 (33.9)	15 (41.7)	
**Age (years)**	44.4 ± 13.4	56.3 ± 16.1	
**Age range**			<0.001
< 50 years	117 (63.9)	13 (36.1)	
50 - 65 years	54 (29.5)	13 (36.1)	
> 65 years	12 (6.6)	10 (27.8)	
**Scholarity in Years**			0.012
0 - 4 years	11 (6)	5 (13.9)	
5 - 8 years	18 (9.8)	9 (25)	

> 8 years	154 (84.2)	22 (61.1)	
**Hospitalization**			<0.001
No	148 (80.9)	16 (44.4)	
Yes	35 (19.1)	20 (55.6)	
Clinical data – post-COVID symptoms			
**Anosmia**			0.002
No	121 (66.1)	33 (91.7)	
Yes	62 (33.9)	3 (8.3)	
**Sleep disorders**			0.848
No	130 (71)	25 (69.4)	
Yes	53 (29)	11 (30.6)	
**Depression**			0.548
No	150 (82)	31 (86.1)	
Yes	33 (18)	5 (13.9)	
**Anxiety symptoms**			0.878
No	135 (73.8)	27 (75)	
Yes	48 (26.2)	9 (25)	
**Headache**			0.598
No	124 (67.8)	26 (72.2)	
Yes	59 (32.2)	10 (27.8)	

Continuous data expressed as mean ± standard deviation. Categorical data expressed as absolute counts and percentages in parentheses. *Chi-square test was used for categorical data and Student's t test for age. CI: Cognitive impairment. Source: Authors elaboration, 2023.

Table S2. Test scores applied in the total group.

Applied test scores	Total group (n=219)
**Median PFEFFER (min – max)**	0 (0 - 30)
**Beck Inventory (IQR)**	0 (0 - 11)

**GDS (IQR)**	3 (0 - 5)
**CDR, median (min – max)**	0 (0 - 3)
**PRMQ, mean ± SD**	7 ± 4
**MMSE, mean ± SD**	26.6 ± 6.1
**ACE-R, mean ± SD**	80.2 ± 20.6
**Orientation/Attention, mean ± SD**	16.1 ± 3.8
**Memory, mean ± SD**	18.7 ± 6.1
**Verbal Fluency, average ± SD**	9.5 ± 3.4
**Language, mean ± SD**	22.8 ± 5.9
**Visual-spatial ability, mean ± SD**	13.2 ± 4

IQR: interquartile range. SD: standard deviation. GDS: Geriatric Depression Scale; CDR: Clinical Dementia Rating; PRMQ: Prospective and Retrospective Memory Questionnaire's; MMSE: Mini-Mental State Examination; ACE-R: Addenbrooke's Cognitive Examination – Revised. Source: Authors elaboration, 2023.

Table S3. Comparison of test scores applied in relation to cognitive status.

	**Normal (n=76)**	**Dementia (n=11)**	**SCD (n=107)**	**MCI (n=25)**	**p***
**Median PFEFFER (min – max)**	0 (0 - 7)	29 (0 - 30)	0 (0 - 26)	0 (0 - 3)	<0.001 [A]
**Beck Inventory (IQR)**	0 (0 - 6)	0 (0 - 0)	2 (0 - 12)	0 (0 - 13)	0.067
**GDS (IQR)**	3.5 (1 – 5.5)	2 (1 - 5)	3 (0 - 5)	4,5(1.5 – 8.5)	0.807
**CDR, median (min – max)**	0 (0 - 0)	3 (0 - 3)	0 (0 - 0)	0 (0 - 1)	<0.001 [A]
**PRMQ, mean ± SD**	6 ± 3	13 ± 8	7 ± 3	7 ± 4	<0.001[A]
**MMSE, mean ± SD**	26.8 ± 6.6	15.2 ± 6.7	27.7 ± 4.9	26.2 ± 2.4	<0.001 [A]
**ACE-R, mean ± SD**	81.6 ± 21.5	36.7 ± 20.1	85.8 ± 16	71.5 ± 6	<0.001 [A,B]
**Orientation/Attention, mean ± SD**	16.2 ± 4.1	8.6 ± 4.5	16.8 ± 3.1	16.2 ± 2	<0.001 [A]
**Memory, mean ± SD**	19.6 ± 6.3	9.2 ± 7.3	20.1 ± 4.7	14.1 ± 3.9	<0.001 [C,D]
**Verbal Fluency, average ± SD**	9.7 ± 3.3	3.2 ± 3	10.4 ± 2.9	7.8 ± 2.5	<0.001 [A,D]
**Language, mean ± SD**	22.9 ± 6.1	10.8 ± 8.6	24.1 ± 4.5	22.2 ± 2.5	<0.001 [A]
**Visual-spatial ability, mean ± SD**	13.4 ± 4.2	4.9 ± 3.6	14.1 ± 3.1	12.4 ± 2.2	<0.001 [A]

IQR: interquartile range. SD: standard deviation. DCS: Subjective cognitive decline; MCI: Mild Cognitive Impairment.

*We used the ANOVA test with Tukey's post-test for means and the Kruskal-Wallis test with multiple comparisons for medians. A: p<0.05 between: "Dementia" vs other groups. B: CCL vs DCS. C: "Dementia" vs DCL and normal. D: CCL vs DCS and normal. GDS: Geriatric Depression Scale; CDR: Clinical Dementia Rating; PRMQ: Prospective and Retrospective Memory Questionnaire's; MMSE: Mini-Mental State Examination; ACE-R: Addenbrooke's Cognitive Examination – Revised. Source: Authors elaboration, 2023.

Table S4. Comparison of test scores applied between normal patients and patients with cognitive decline.

	Normal (n=76)	CD (n=143)	p
**Median PFEFFER (min – max)**	0 (0 - 7)	0 (0 - 30)	0.144
**Beck Inventory (IQR)**	0 (0 - 6)	2 (0 - 12)	0.048
**GDS (IQR)**	3.5 (1 - 5,5)	3 (0 - 5)	0.790
**CDR, median (min – max)**	0 (0 - 0)	0 (0 - 3)	0.013
**PRMQ, mean ± SD**	6 ± 3	7 ± 4	0.084
**MMSE, mean ± SD**	26.8 ± 6.6	26.5 ± 5.8	0.69
**ACE-R, mean ± SD**	81.6 ± 21.5	79.5 ± 20.2	0.465
**Orientation/Attention, mean ± SD**	16.2 ± 4.1	16.1 ± 3.7	0.855
**Memory, mean ± SD**	19.6 ± 6.3	18.2 ± 5.9	0.106
**Verbal Fluency, average ± SD**	9.7 ± 3.3	9.4 ± 3.5	0.593
**Language, mean ± SD**	22.9 ± 6.1	22.8 ± 5.8	0.868
**Visual-spatial ability, mean ± SD**	13.4 ± 4.2	13.1 ± 3.9	0.626

*Student's t test was used to compare means, and the Mann-Whitney test to compare medians. IQR: interquartile range. SD: standard deviation. DC: Cognitive Decline. GDS: Geriatric Depression Scale; CDR: Clinical Dementia Rating; PRMQ: Prospective and Retrospective Memory Questionnaire's; MMSE: Mini-Mental State Examination; ACE-R: Addenbrooke's Cognitive Examination – Revised. Source: Authors elaboration, 2023.

Table S5. Comparison of test scores applied between patients with and without cognitive impairment.

	No cognitive impairment (n=183)	CI (n=36)	p*

**Median PFEFFER (min – max)**	0 (0 - 26)	0 (0 - 30)	<0.001
**Beck Inventory (IQR)**	0 (0 - 11)	0 (0 - 12)	0.785
**GDS (IQR)**	3 (0 - 5)	3 (1 - 6)	0.69
**CDR, median (min – max)**	0 (0 - 0)	0 (0 - 3)	<0.001
**PRMQ, mean ± SD**	6 ± 3	9 ± 6	0.033
**MMSE, mean ± SD**	27.4 ± 5.7	22.9 ± 6.6	<0.001
**ACE-R, mean ± SD**	84.1 ± 18.5	60.9 ± 20.1	<0.001
**Orientation/Attention, mean ± SD**	16.6 ± 3.5	13.9 ± 4.6	<0.001
**Memory, mean ± SD**	19.9 ± 5.4	12.6 ± 5.6	<0.001
**Verbal Fluency, average ± SD**	10.1 ± 3.1	6.4 ± 3.4	<0.001
**Language, mean ± SD**	23.6 ± 5.2	18.7 ± 7.3	<0.001
**Visual-spatial ability, mean ± SD**	13.8 ± 3.6	10.1 ± 4.4	<0.001

*Student's t test was used to compare means, and the Mann-Whitney test to compare medians. IQR: interquartile range. SD: standard deviation. CC: Cognitive impairment. GDS: Geriatric Depression Scale; CDR: Clinical Dementia Rating; PRMQ: Prospective and Retrospective Memory Questionnaire's; MMSE: Mini-Mental State Examination; ACE-R: Addenbrooke's Cognitive Examination – Revised. Source: Authors elaboration, 2023.

Table S6. Comparison of APOE genotypes and their alleles in relation to cognitive status.

	**Normal (n=76)**	**Dementia (n=11)**	**SCD (n=107)**	**MCI (n=25)**	**p**
APOE					0.923
E2/E2	0 (0)	0 (0)	1 (1,1)	0 (0)	
E2/E3	5 (8.2)	1 (9.1)	6 (6.7)	2 (8.7)	
E2/E4	0 (0)	0 (0)	1 (1,1)	0 (0)	
E3/E3	46 (75.4)	6 (54.5)	55 (61.1)	15 (65.2)	
E3/E4	9 (14.8)	4 (36.4)	25 (27.8)	5 (21.7)	
E4/E4	1 (1.6)	0 (0)	2 (2.2)	1 (4.3)	
**Alleles**					

**E2**					0.999
No	56 (91.8)	10 (90.9)	82 (91.1)	21 (91.3)	
Yes	5 (8.2)	1 (9.1)	8 (8.9)	2 (8.7)	
**E3**					0.618
No	1 (1.6)	0 (0)	4 (4.4)	1 (4.3)	
Yes	60 (98.4)	11 (100)	86 (95.6)	22 (95.7)	
**E4**					0.190
No	51 (83.6)	7 (63.6)	62 (68.9)	17 (73.9)	
Yes	10 (16,4)	4 (36,4)	28 (31,1)	6 (26,1)	

Categorical data expressed as absolute counts and percentages in parentheses. The chi-square or Fisher's exact test was used. SCD: Subjective cognitive decline; MCI: Mild Cognitive Impairment; APOE: Apolipoprotein E. Source: Authors elaboration, 2023.

Table S7. Comparison of APOE genotypes and alleles between groups with and without cognitive impairment.

	**No cognitive impairment (n=151)**	**CI (n=34)**	**p**
**APOE**			0.966
E2/E2	1 (0.7)	0 (0)	
E2/E3	11 (7.3)	3 (8.8)	
E2/E4	1 (0.7)	0 (0)	
E3/E3	101 (66.9)	21 (61.8)	
E3/E4	34 (22.5)	9 (26.5)	
E4/E4	3 (2)	1 (2.9)	
**Alleles**			
**E2**			0.968
No	138 (91.4)	31 (91.2)	

Yes	13 (8.6)	3 (8.8)	
**E3**			1.000
No	5 (3.3)	1 (2.9)	
Yes	146 (96.7)	33 (97.1)	
**E4**			0.610
No	113 (74.8)	24 (70.6)	
Yes	38 (25.2)	10 (29.4)	

Categorical data expressed as absolute counts and percentages in parentheses. The chi-square or Fisher's exact test was used. CI: Cognitive impairment; APOE: Apolipoprotein E. Source: Authors elaboration, 2023.
